# Evaluation of 2 Artificial Intelligence Software for Chest X-Ray Screening and Pulmonary Tuberculosis Diagnosis: Protocol for a Retrospective Case-Control Study

**DOI:** 10.2196/36121

**Published:** 2023-07-25

**Authors:** Muhammad Faiz Mohd Hisham, Noor Aliza Lodz, Eida Nurhadzira Muhammad, Filza Noor Asari, Mohd Ihsani Mahmood, Zamzurina Abu Bakar

**Affiliations:** 1 Institute for Public Health National Institute of Health Ministry of Health Malaysia Shah Alam Malaysia; 2 Sector of Tuberculosis & Leprosy Disease Control Division Ministry of Health Malaysia Putrajaya Malaysia; 3 Respiratory Medicine Institute Ministry of Health Malaysia Kuala Lumpur Malaysia

**Keywords:** artificial intelligence, AI, evaluation, pulmonary tuberculosis, PTB, chest x-ray, CXR, screening

## Abstract

**Background:**

According to the World Bank, Malaysia reported an estimated 97 tuberculosis cases per 100,000 people in 2021. Chest x-ray (CXR) remains the best conventional method for the early detection of pulmonary tuberculosis (PTB) infection. The intervention of artificial intelligence (AI) in PTB diagnosis could efficiently aid human interpreters and reduce health professionals’ work burden. To date, no AI studies have been evaluated in Malaysia.

**Objective:**

This study aims to evaluate the performance of Putralytica and Qure.ai software for CXR screening and PTB diagnosis among the Malaysian population.

**Methods:**

We will conduct a retrospective case-control study at the Respiratory Medicine Institute, National Cancer Institute, and Sungai Buloh Health Clinic. A total of 1500 CXR images of patients who completed treatments or check-ups will be selected and categorized into three groups: (1) abnormal PTB cases, (2) abnormal non-PTB cases, and (3) normal cases. These CXR images, along with their clinical findings, will be the reference standard in this study. All patient data, including sociodemographic characteristics and clinical history, will be collected prior to screening via Putralytica and Qure.ai software and readers’ interpretation, which are the index tests for this study. Interpretation from all 3 index tests will be compared with the reference standard, and significant statistical analysis will be computed.

**Results:**

Data collection is expected to commence in August 2023. It is anticipated that 1 year will be needed to conduct the study.

**Conclusions:**

This study will measure the accuracy of Putralytica and Qure.ai software and whether their findings will concur with readers’ interpretation and the reference standard, thus providing evidence toward the effectiveness of implementing AI in the medical setting.

**International Registered Report Identifier (IRRID):**

PRR1-10.2196/36121

## Introduction

Tuberculosis is a contagious disease that leads to substantial morbidity and mortality worldwide. It is caused by the *Mycobacterium tuberculosis* bacteria (MTB). Tuberculosis is spread from infected people to others through the air. Even though tuberculosis is curable and preventable, the World Health Organization (WHO) estimated that 10 million people contracted tuberculosis and 1.6 million people died from it worldwide in 2021 [1]. In Malaysia, the incidence of tuberculosis is alarming, where 21,186 cases were reported in 2021 with an estimated 97 cases per 100,000 people, which is growing at an average annual rate of 1.96% [2].

The diagnosis of active tuberculosis is made either by skin test, blood test, chest x-ray (CXR), sputum acid-fast bacilli (AFB) smear and culture, polymerase chain reaction, or a combination of these tests. CXR remains the best conventional method for early detection of pulmonary tuberculosis (PTB) infection and the most recommended method for tuberculosis prevalence survey due to its high sensitivity [3]. The specificity of CXR for PTB could be more significant than evaluating symptoms alone, depending on how the CXR is read. Triage using CXR will also help to minimize the number of individuals undergoing bacteriological tuberculosis testing without reducing the detection of true tuberculosis cases [4]. Despite numerous advantages, the lack of qualified radiologists who can interpret CXR images, especially in high–tuberculosis burden countries, and the high inter- and intrareader variability are among the substantial drawbacks of implementing CXR as one of the diagnostic tools [4]. This leads to the impairment of screening efficacy and scale-up efforts [4,5]. Thus, the introduction of artificial intelligence (AI) in medical imaging with multiple technological approaches aided in reducing those obstacles [6,7].

AI is the ability of a digital computer or machine to perform tasks commonly associated with intelligent beings [8]. The rapid development of information technology worldwide and its use in multidiscipline practice make AI a new area of interest, especially in the medical profession [9]. Recently, approaches to using innovation in the health care system to support patient care are highly sought-after comorbidities. Thus, with AI technology, medical imaging (digital x-ray) can reduce the period of tuberculosis management, and patients with tuberculosis can receive early treatment. At present, various AI methodologies for the diagnosis of tuberculosis are being developed, that is, general regression neural network, multilayer neural network, convolutional neural network (CNN), artificial immune system, fuzzy logic controller, and real-world tournament selection, all of which have yielded promising accuracy for tuberculosis detection [10]. The capability of AI to recognize tuberculosis-related abnormalities via CXR, especially in mass screening with minimal time, has substantially improved and outperformed the human interpreter, thus reducing the work burden of health professionals [11]. Several studies have evaluated the efficacy and accuracy of using AI worldwide [12], but no such study has been carried out in Malaysia.

Therefore, this study aims to evaluate the performance of Putralytica and Qure.ai software for CXR screening and PTB diagnosis among the Malaysian population.

## Methods

### Sampling Design

This is a retrospective case-control AI software evaluation study that will take place at 3 different health facilities: the Respiratory Medicine Institute, National Cancer Institute, and Sungai Buloh Health Clinic. The study is expected to take 1 year to complete. We will collect CXR images from all adults (≥15 years) who meet the inclusion criteria for this study (Textbox 1). A total of 1500 CXR images will be retrieved; 500 of which will be abnormal PTB cases, 500 of which will be abnormal non-PTB cases, and 500 of which will be normal cases (healthy control). In this study, these images will serve as the reference standard. Primary demographic data from the CXR reports (patient’s age, gender, and race), patients’ clinical investigations and medical history (symptoms, CXR interpretation, and comorbidity), and laboratory results (AFB smear, MTB culture, and GeneXpert assay) will be collected by assessing electronic or hard-copy medical records.

**Inclusion criteria.**

**Inclusion criteria for abnormal pulmonary tuberculosis (PTB) cases**

- Aged 15 years and older
- One chest x-ray (CXR) per PTB case or mix case with other lung pathology who completed treatment for tuberculosis
- One CXR per patient upon diagnosis
- Spectrum of severity is reflective of real clinical setting

**Inclusion criteria for abnormal non-PTB cases**

- Aged 15 years and older
- One CXR per abnormal case (non-PTB case)
- One CXR per patient upon diagnosis
- Spectrum of disease(s) is reflective of real clinical setting

**Inclusion criteria for normal cases (healthy control)**

- Aged 15 years and older
- One CXR per healthy control
- Latest CXR image is used

### Sample Size Calculation

According to a sample size estimation when comparing sensitivities (Sn) or specificities (Sp) in a matched-groups diagnostic study in radiology research, a total of 1500 CXR images are needed in this study. It was calculated with the assumption that the probability of disagreement between both AI software is 0.131, with the highest Sp/Sn being 0.95 and the lowest Sp/Sn being 0.91, a power of 80%, and significant level of 5%:




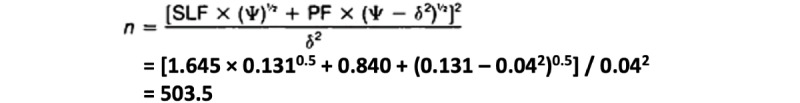




### Work Flowchart

**Figure 1:**
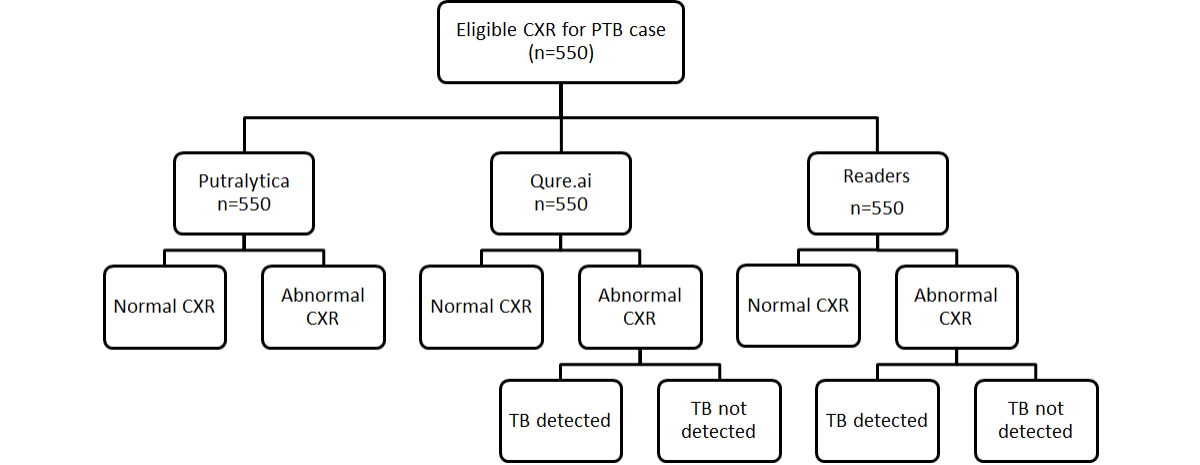
Work flowchart for chest x-ray (CXR) images of abnormal pulmonary tuberculosis (PTB) cases. TB: tuberculosis.

**Figure 2:**
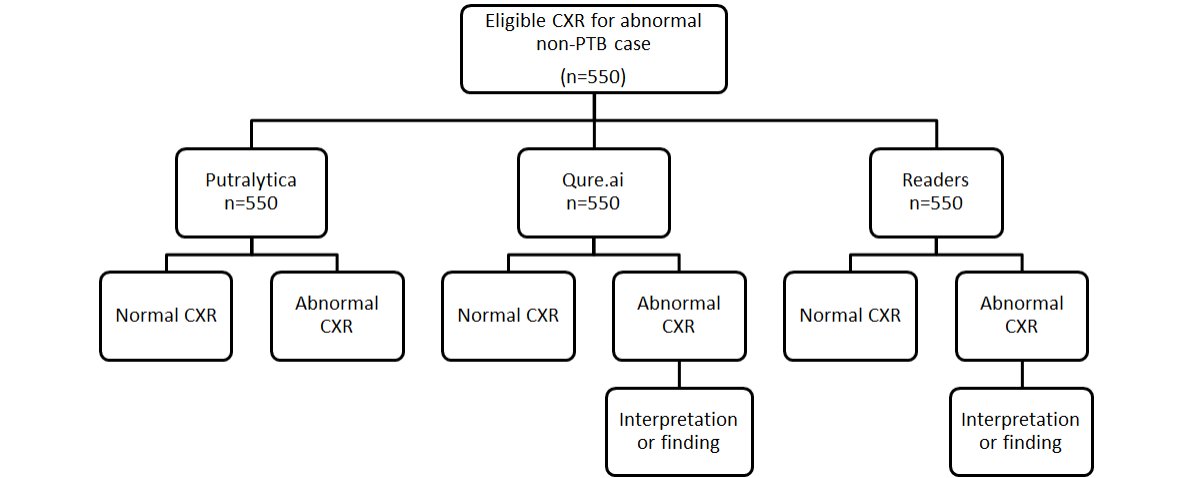
Work flowchart for chest x-ray (CXR) images of abnormal non-PTB cases. PTB: pulmonary tuberculosis.

**Figure 3:**
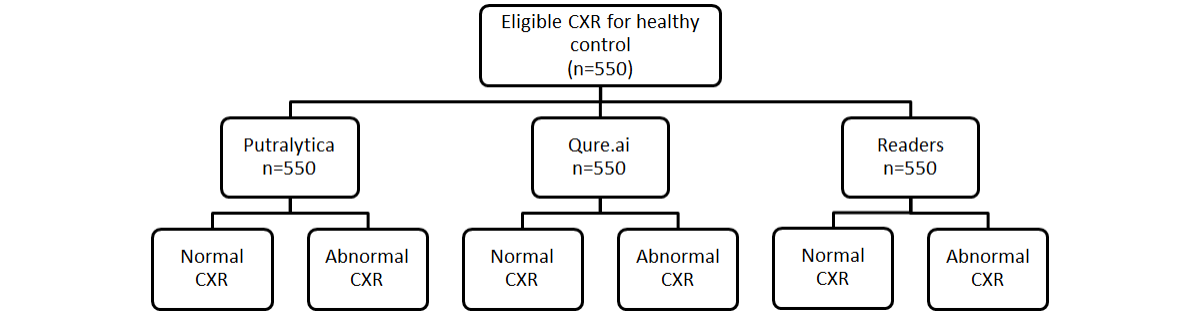
Work flowchart for chest x-ray (CXR) images of normal cases (healthy control).

The reporting of this study will adhere to the Standard for the Reporting of Diagnostic Accuracy Studies (STARD) guidelines. The work flowchart for CXR images of each case is shown in Figures 1-3.

### Reference Standard

The reference standard in this study is the interpretation of the preselected CXR images according to the 3 aforementioned categories. First, an abnormal PTB case is defined as a patient with PTB who had been prescribed and completed PTB treatment based on the following criteria: formal diagnosis; symptom status; CXR interpretation; and results from laboratory investigation, including AFB smear, MTB culture, and GeneXpert assay. Second, an abnormal non-PTB case is defined as any patient with lung abnormalities that are not PTB, based on the following criteria: formal diagnosis, symptom status, and CXR interpretation. Third, a healthy control is defined as any healthy person with normal CXR, including health screening and contact with a person with PTB who completed follow-up, based on the following criteria: formal diagnosis and CXR interpretation.

### Index Test

#### Qure.ai (India)

Qure.ai is a CXR screening tool that detects signs of pulmonary, hilar, and pleural tuberculosis. The AI algorithm underlying Qure.ai is trained to detect normal and abnormal CXR images, classical primary PTB, and atypical manifestations. The software is trained and validated using a data set of over 2.5 million CXR images worldwide. Qure.ai classifies findings from the CXR into 3 categories: active tuberculosis, nonactive tuberculosis and other lung or chest abnormalities, and normal. The final interpretation generated by this software is either PTB or not PTB, together with a conclusive radiological finding for abnormalities other than PTB. The AI algorithms produce a continuous abnormality for threshold scoring (from 0 to 1), representing the probability of the presence of tuberculosis. It claims to yield an accuracy of >90%, with the established threshold of 0.5 by the AI developer. The software can be accessed with or without an internet connection.

#### Putralytica (Malaysia)

Putralytica is a web-based platform that uses machine learning and automatic detection of pathology. This AI software is a type of CNN that can interpret normal and abnormal CXR only. The CNN was trained and internally validated using an open-access public CXR data set from the National Institutes of Health containing 112,120 frontal view CXR images. The software was developed using the Python 3 language with the KERAS and Tensorflow 2.0 application programming interface framework. The AI algorithms produce a continuous abnormality for threshold scoring (from 0 to 100), representing the probability of the CXR abnormality. It claims to yield an accuracy of >85%, with the established threshold of 60% by the AI developer. The software can be accessed with or without an internet connection.

#### Readers’ Interpretation

The readers consist of 3 professionals with varying levels of experience in the radiology field: a clinical radiologist with >10 years of service, a clinical radiologist with <10 years of service, and a medical officer who works in the radiology department. These readers will be oblivious to clinical and sociodemographic information about patients, and they will analyze the CXR images independently. They will fill out a structured form to provide their interpretation based on the given variables.

### Case Definition

#### Clinically Diagnosed Tuberculosis

A clinically diagnosed tuberculosis case is a patient who does not fulfil the criteria for bacteriological confirmation but has been diagnosed with active tuberculosis by a clinician or other medical practitioner, who has decided to give the patient an entire course of tuberculosis treatment. This definition includes cases diagnosed with x-ray abnormalities or suggestive histology and extrapulmonary cases without laboratory confirmation. Clinically diagnosed cases subsequently found to be bacteriologically positive (before or after starting treatment) should be reclassified as bacteriologically confirmed [13].

#### Bacteriologically Confirmed Tuberculosis

A bacteriologically confirmed tuberculosis case is a patient from which a biological specimen is positive by smear microscopy, culture, or WHO-recommended rapid diagnostic tool such as the GeneXpert MTB/RIF assay. All such cases should be notified, regardless of whether tuberculosis treatment has started [13].

### Working Definition

In this study, a PTB case is defined as an abnormal CXR (suggestive of PTB), clinical finding with a formal diagnosis suggestive of PTB, a laboratory investigation suggestive of the presence of tuberculosis, or any combination of these. An abnormal non-PTB case is defined as any abnormal CXR excluding the PTB cases, and a normal case for healthy control is defined as any normal CXR finding.

### Data Collection and Data Handling

A structured form will be designed to retrieve and collate data from the existing reports of CXR examination and laboratory investigation with AI software–interpreted results and the readers’ interpretation. The information will be kept in an assigned documented file where it can only be accessed by the investigators. Considering the duration of this research project and to allow the process of publishing the findings, the research data will be stored and archived for 18 months. Afterward, the research data will be destroyed by permanently deleting the file from the storage hard disk.

### Ethics Approval

Ethics approval had been granted by National Medical Research Register and Medical Research and Ethics Committee. Permission to conduct the study is upon approval by the State Health Director.

### Privacy and Confidentiality

For all data, privacy and confidentiality will be maintained. The result of the study will be anonymized. The information revealed will not allow the identification of any individual participants. The result will also be in an aggregated form and will not identify any individual or address. Only those involved with this study and data analysis will have access to the data.

### Dissemination

The analysis results will be presented in technical or scientific meetings at the state or Ministry of Health level and published as technical reports or journal articles. Details of individuals will not be disclosed but will only be reported in an aggregated form.

### Statistical Analysis

All retrieved data will be downloaded and exported to the SPSS software (IBM Corp) for analysis. Data will be grouped based on the variable, description, and data type to facilitate analysis (Table 1). The results of CXR interpretation from both software and the readers will be specified solely based on the description provided (Table 2). This is done to avoid lengthy reader responses and ensure interpretation consistency and uniformity. Descriptive analysis will be used to describe the sociodemographic and clinical characteristics of the patients. Data analysis will be performed for both AI software against the reference standard by constructing 2 × 2 tables to determine true positive, false positive, false negative, and true negative results. The overall diagnostic accuracy parameters and their 95% CIs will be computed: sensitivity and specificity, positive likelihood ratio, negative likelihood ratio, Youden index, and area under the curve. Cohen κ statistic will be applied to determine interrater agreement among the readers and between the readers’ and the AI software’s interpretations.

**Table 1:** Patients’ medical history and laboratory investigation.

Variable and definition		Description	Data type
**1. Sociodemographic information**			
	Research ID	Unique coding	String
	Ethnicity among Malaysian patients	1=Malay 2=Chinese 3=Indian 4=Bumiputera Sabah 5=Bumiputera Sarawak 6=Others (specify)	Categorical
	Date of birth (≥15 years)	Exact date of birth (dd/mm/yyyy)	String
	Sex	1=Male 2=Female	Categorical
**2. Clinical background**			
	Date of admission	N/A^a^	String
	Date of CXR^b^ taken	N/A	String
	Formal diagnosis	N/A	String
	Comorbidities	1=Diabetes 2=Hypertension 3=Hypercholesterolemia 4=History of tuberculosis 5=HIV 6=Smoking 7=Asthma 8=COPD^c^ 9=Bronchiectasis 10=Chronic bronchitis 11=Heart failure 12=Pulmonary fibrosis 13=Others (specify)	Categorical
	Tuberculosis-like symptoms status	1=Coughing for >2 weeks (prolonged cough) 2=Coughing up mucus 3=Coughing up blood 4=Fever (prolonged fever) 5=Weight loss 6=Night sweat	Categorical
**3. Laboratory investigation**			
	Sample type	1=Sputum 2=Aspirate 3=BAL^d^ 4=BW^e^ 5=PF^f^ 6=Others (specify)	Categorical
	Date of direct smear	N/A	String
	Direct smear	1=Positive (3+) 2=Positive (2+) 3=Positive (1+) 4=Negative 5=Not done	Categorical
	Date of MTB^g^ culture	N/A	String
	MTB culture	1=Growth (MTB) 2=No growth 3=Non-MTB (specify) 4=Not done	Categorical
	Date of GeneXpert assay	N/A	String
	GeneXpert assay	1=Detected (High, RIF^h^ resistance) 2=Detected (High) 3=Detected (Med, RIF resistance) 4=Detected (Med) 5=Detected (Low, RIF resistance) 6=Detected (Low) 7=Not detected 8=Not done	Categorical

^a^N/A: not applicable.

^b^CXR: chest x-ray.

^c^COPD: chronic obstructive pulmonary disease.

^d^BAL: bronchoalveolar lavage.

^e^BW: bronchial wash.

^f^PF: pleural fluid.

^g^MTB: *Mycobacterium tuberculosis* bacteria.

^h^RIF: rifampin.

**Table 2:** Chest x-ray (CXR) interpretation.

Variable name	Definition	Description	Data type
1. CXR interpretation for abnormalities (PTB^a^ and non-PTB related)	Results for: Qure.ai Radiologist	1=Consolidation 2=Cavity lesion 3=Nodule (tuberculoma) 4=Pleural effusion 5=Hilar 6=Miliary tuberculosis 7=Discrete nodule (without calcification) 8=Discrete nodules with retraction 9=Discrete fibrotic scar, line opacity, or reticular densities 10=Discrete fibrotic scar with retraction 11=Cardiac enlargement or anomalies 12=Pulmonary abnormality (mass) 13=Pleural thickening irregularities 14=Blunting 15=Granuloma 16=Other	Categorical
2. CXR interpretation (basic)	Results for: Putralytica Qure.ai Radiologist	1=Abnormal (active tuberculosis) 2=Abnormal (nonactive tuberculosis) 3=Abnormal (not tuberculosis) 3=Abnormal 4=Normal	Categorical

^a^PTB: pulmonary tuberculosis.

## Results

Data collection on sites is expected to commence in August 2023, once the funding has been granted. It is anticipated that 1 year will be needed to conduct the study. Data collection will commence in phase 1 of the project (3-5 months), whereas CXR image distribution for readers’ interpretation will take place in phase 2 of the project (3-6 months). All results will be collated and presented at the state and ministry levels. Reports and manuscripts will be produced throughout 2024 after obtaining approval and resolution from all parties involved.

## Discussion

This study considers biases commonly overlooked by researchers, particularly in diagnostic accuracy studies. To reduce the spectrum of bias (disease and nondisease), this study will include a variety of CXR abnormalities, not only to detect the presence of PTB but also other abnormalities, with CXR images chosen based on severity level. All CXR images will be in the form of digitalized images to standardize the methodology and reduce measurement bias. Most studies only focused on software performance or the agreement between radiological interpretations of CXR image results independently. Some studies do not use MTB culture and GeneXpert assay as bacteriological evidence in their reference standard, and some do not even compare the performance of the AI with human readers. This study, indeed, will incorporate all variables and analyses into a single comprehensive assessment. The difficulty we encountered while conducting this study was the need to visit several sites to collect CXR images for use as the standard reference. We had to adjust various processes and workflow to get the patients’ information without interfering with the staff’s primary responsibilities at the study sites. In the future, this study will be instrumental in proving that AI is appropriate when there are various obstacles, whether in clinical settings or fieldwork. In clinical settings, AI can alleviate the burden on medical personnel, particularly in areas where imaging technology is widely used with large workloads. In fieldwork, AI is very beneficial for prevalence studies involving data collection in remote areas and studies that require interpreting numerous results quickly and accurately.

## Abbreviations

AFB: acid-fast bacilli
AI: artificial intelligence
CNN: convolutional neural network
CXR: chest X-ray
MTB: Mycobacterium tuberculosis bacteria
PTB: pulmonary tuberculosis
STARD: Standard for the Reporting of Diagnostic Accuracy Studies
WHO: World Health Organization
